# Characterization and In Vitro Digestion Kinetics of Purified Pulse Starches: Implications on Bread Formulation

**DOI:** 10.3390/foods14020328

**Published:** 2025-01-20

**Authors:** Oluwatoyin O. Sangokunle, Sarah G. Corwin, Bruce R. Hamaker

**Affiliations:** 1 Department of Food Science, College of Agriculture and Food Sciences, Florida A and M University, Tallahassee, FL 32307, USA; 2 Whistler Center for Carbohydrate Research and Department of Food Science, Purdue University, West Lafayette, IN 47907, USA; sarahgcorwin@gmail.com (S.G.C.); hamakerb@purdue.edu (B.R.H.)

**Keywords:** starch, pulses, bread, peas, ingredient formulation

## Abstract

This study investigated the contribution of pulse starches (PSs) to the slowly digestible starch (SDS) properties observed in pulses. Purified pulse starches from 17 commonly consumed pulses were examined, focusing on their digestion kinetics using a pancreatic alpha-amylase (PAA) and rat intestinal acetone powder (RIAP) mixture. Chickpea starch, exhibiting a slow digestibility profile, was incorporated as an ingredient to confer slow digestibility to refined wheat flour bread. Our findings reveal that some PSs exhibited low digestibility when gelatinized (100 °C, 30 min) and retrograded (7 days, 4 °C). Rapid retrogradation was observed in starch from chickpeas, lentils, field peas, adzuki beans, navy beans, large lima beans, and great northern beans. The incorporation of chickpea starch into fortified bread significantly improved its slow digestibility properties. This study reveals the potential of pulse starch as a promising functional ingredient for baked products, related to the faster retrogradation of many pulse-sourced starches. These findings contribute valuable insights into the slow digestibility attributes of pulse starches for developing food products with enhanced nutritional profiles.

## 1. Introduction

Pulses have gained recognition for their association with low glycemic response, serving as a crucial component of a health-promoting diet. Pulses are nutritionally dense, with their protein content exceeding 20–30%, starch comprising more than 50%, and a balance of soluble and insoluble fibers constituting up to 10% of their seed weight [1,2]. The health benefits attributed to pulse consumption, particularly in promoting a low glycemic response, are closely linked to the high content of slowly digestible starch (SDS), resistant starch (RS), and dietary fibers, along with significant amounts of bioactive polyphenols [3,4].

With growing consumer interest in diets high in fiber, low in sugar, and rich in protein, the application of SDS and RS in processed foods has risen. These starches contribute to a lower glycemic response and enhance gut health through fermentation by the gut microbiota, a factor increasingly recognized for its role in metabolic health [4]. With their high levels of SDS and RS, pulses meet the rising demand for functional foods that offer nutritional benefits and potential health improvements, such as improving the quality of rapidly digestible carbohydrate products like white wheat bread [5].

White wheat bread, often used as a reference for high glycemic response due to its quick conversion to simple sugars [6], poses risks for individuals with type-2 diabetes and other cardiometabolic conditions. Increasing consumer awareness of the drawbacks of high-glycemic and high-sugar diets has led to a demand for foods with enhanced nutritional quality and functional properties. With their slowly digestible starches, pulses offer a promising solution to reduce these risks. Here, we investigated the potential of purified pulse starch as a functional, slowly digestible ingredient, aiming to improve the nutritional profile of white wheat bread and contribute to human health.

## 2. Materials and Methods

### 2.1. Materials

Purified pulse starches of known proximate compositions were used, as previously reported by [7]. A resistant starch assay kit, amylose/amylopectin, high-amylose maize, glucose standard, and total starch reagent kit were obtained from Megazyme, Bray, Ireland. Maltodextrin, corn starch (standard), pancreatic alpha-amylase/amyloglucosidase (PAA/AMG), rat intestinal acetone powder (RIAP), and ampicillin were obtained from Sigma Aldrich (MO, USA). All other chemical reagents were of analytical grade.

### 2.2. Starch Source

Purified pulse starches were obtained from adzuki bean, black bean, black-eyed pea, cranberry bean, field pea, chickpea pea, great northern bean, large lima, lentil, light red kidney, navy bean, Nigerian honey bean, pink bean, pinto bean, small red bean, small white bean, and whole moth bean. The pulses were purchased from local grocery stores and are reported elsewhere [7]. As previously reported, the pulse starches were isolated and purified using the wet-milling method to produce a quality-grade starch with less soluble protein, total lipids, and total ash [7].

### 2.3. Scanning Electron Microscopy

Pulse starch granule surfaces were analyzed using a scanning electron microscope (SEM, FEI NovaNano, FEI Company, Hillsborough, OR, USA). Starch granules were attached to a carbon tape, coated using platinum, and analyzed using SEM at 5 kV.

### 2.4. Thermal and Structural Analysis

#### 2.4.1. Differential Scanning Calorimetry

Differential scanning calorimetry (DSC) was performed on pulse starch with water, using a Q2000 DSC (TA instruments, New Castle, DE, USA), following the protocol outlined by Dong and Vasanthan, 2020. Starches were weighed in an aluminum pan with water added in a 1:3 ratio and sealed for starch gelatinization. The samples were equilibrated at 25 °C for 24 h. An empty reference pan was heated with the sample pan from 0 to 110 °C at 10 °C/min using calibrated DSC. The gelatinization enthalpy was measured from the melting peak observed from the thermograms and integrated using the TA Universal Analysis Software (https://www.tainstruments.com/). A similar process was also performed to study the amylose retrogradation during 14 days of storage at 4 °C. All measurements were performed in triplicate.

#### 2.4.2. Molecular Weight Distribution of Amylopectin

For the debranching step, briefly, starch was added to DMSO/LiBr (0.5% *w*/*w*) and heated in a thermomixer (80 °C, 350 rpm, 24 h) with intermittent inversion by hand to ensure complete dislodging of the starch samples from the Eppendorf tube. Samples were centrifuged (4000× *g* for 10 min) to remove excess DMSO/LiBr. Starch was immediately dispersed in deionized water and then heated in boiling water for complete solids dispersion (<20 min). The dispersion was cooled (25 °C, 20 min), and acetate buffer (0.1 M, pH 3.5), sodium azide solution (0.04 g/L), and isoamylase solution (2.5 µL, Megazyme, Wicklow, Ireland) were added, respectively. The mixture was incubated (37 °C, 3 h) and neutralized using 0.1 mL NaOH. The sample was freeze-dried for 24 h, dissolved in DMSO (4 mg/mL), vacuum-filtered (0.25 µM), transferred into size-exclusion chromatography (SEC) vials, and injected into the SEC system (Agilent 1260 series, Agilent Technologies, Waldbronn, Germany) equipped with a refractive index detector (RID, 1260 RID, Agilent, Agilent Technologies, Waldbronn, Germany). The area under the curve of the SEC was obtained and used to calculate the amylose content of the purified pulse starches from the amylose and amylopectin fractions.

### 2.5. In Vitro Digestion

#### Digestion of Pulse Starch Using Ground and Sieved RIAP with Pancreatic Alpha-Amylase

Gelatinized and retrograded pulse starches were digested using the method of [8] with slight modifications. Starch (120 mg) was mixed with phosphate buffer (100 MM, 1% starch final concentration) and stored at −80 °C in microcentrifuge tubes until ready. The buffered starch solution was gelatinized (100 °C, 30 min, 1300 RPM) using a thermomixer (Eppendorf, Thermomixer C, Hampton, NH, USA). Similarly, the process was repeated for starch retrogradation, and then the starch was frozen (7 days and stored for an additional 7 days at 4 °C). Following the temperature treatments, the starches were pre-incubated at 37 °C in a water bath (600 RPM for 10 min). A total of 900 μL of enzyme suspension (5% RIAP and 50 U/mL PAA, 0.005% ampicillin at pH 6.7) was added to each tube and returned to the water bath at 37 °C and 600 RPM. Aliquots of 110 mL of digested substrate were transferred into new microtubes at intervals (15, 30, 45, 60, 120, and 180 min), and enzymes were inactivated at 100 °C for 5 min in a shaking water bath. The digested substrate was diluted tenfold at the end of inactivation and cooling. The glucose release was measured using a D-glucose oxidase/peroxidase (GOPOD) assay kit (Megazyme, Bray, Ireland) and read at 510 nanometers using a SpectraMax 190 Absorbance Microplate Reader spectrophotometer (San Jose, CA, USA).

### 2.6. Bread Making

The description of the bread recipe is summarized in Supplementary Table S1. In total, 50% chickpea flour (GF) (recipe 001) and 13% chickpea starch (GS) of the total weight of ingredients were added (recipe 002) to the total ingredients used to make bread, respectively, and compared to commercially available regular white wheat bread. After baking (40 min, 350 °C) and cooling (25 °C for 45 min), the bread samples were milled into a uniform particle size (<6 mesh) in a commercial blender, homogenized, and stored refrigerated.

#### In Vitro Bread Digestion

According to the method, the nutritional classification and percentage digestibility were characterized using a Megazyme digestible and resistant starch assay kit (Bray, Ireland) [9]. Similarly, the bread digestibility was determined for starch retrogradation and the effect on digestibility at 14 days of storage at 4 °C (bread was stored at 4 °C for 14 days). A slice of bread was randomly selected and used to determine the rate of digestion at days 0, 3, 7, and 14. Similarly, digestion kinetics were analyzed for glucose release at 20, 60, 120, 180, and 240 min using a Megazyme D-glucose assay kit (Bray, Ireland). The laboratory-made bread was compared to commercial white wheat bread.

## 3. Results and Discussion

### 3.1. Scanning Electron Micrographs of Purified Pulse Starches

**Figure 1:**
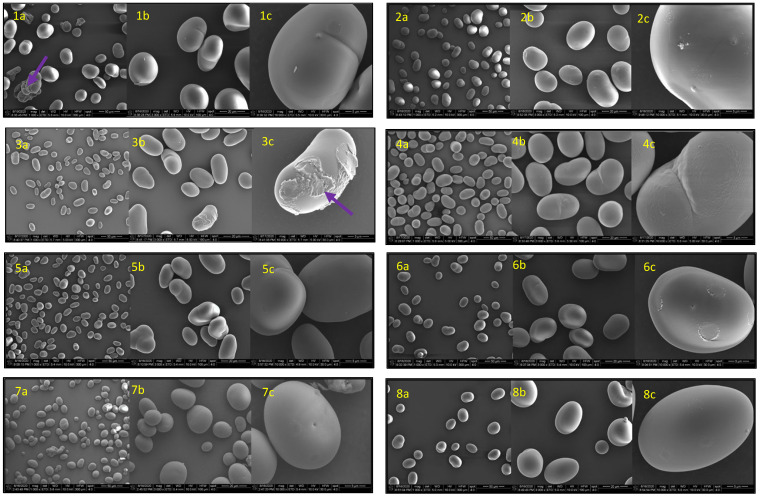

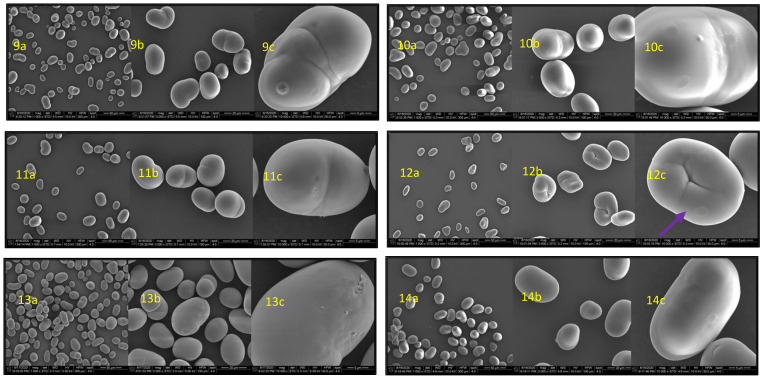
Scanning electron micrographs of purified pulse starches of (**1a**–**1c**), adzuki bean; (**2a**–**2c**), black bean; (**3a**–**3c**), black-eyed pea; (**4a**–**4c**), cranberry bean; (**5a**–**5c**), field pea; (**6a**–**6c**), chickpea; (**7a**–**7c**), great northern bean; (**8a**–**8c**), large lima bean; (**9a**–**9c**), lentil; (**10a**–**10c**), light red kidney bean; (**11a**–**11c**), navy bean; (**12a**–**12c**), Nigerian honey bean; (**13a**–**13c**), pink bean; (**14a**–**14c**), pinto bean. a, b, and c indicate 1000×, 3000×, and 10,000× magnifications and represent a scale of 50, 20, and 5 µm, respectively.

In a previous study [7], pulse starches were isolated, purified, and characterized, which included light microscopy examinations. In this study, we further characterized the surface morphology of the pulse starch granules using SEM. The micrographs (Figure 1(1a–14c)) show distinct characteristics across the various pulse starches, emphasizing their unique features.

The SEM images of the pulse starches illustrate granules devoid of surface pores, lipids, and residual protein, aligning with the findings of previous studies [10,11]. The absence of surface pores is a crucial attribute associated with the slow digestion and high resistant starch (RS) content of raw purified pulse starches. The lack of pores slows the accessibility of enzymes to the starch internal structures. Interestingly, the adzuki bean and black-eyed pea granules exhibited surface bruises (Figure 1(1a,3c)), an observation linked to the specific isolation and purification conditions employed in our methodology [7].

A distinctive observation emerged for the Nigerian honey bean (cowpea) starch granules, where a beany shape with grooves and fissures (as indicated by the arrow in Figure 1(12c)) was more pronounced than with the other purified pulse starches. Generally, these starches exhibited various shapes, including round, oval, irregular, and beany, with varying sizes. This supports our previous characterization using light microscopy [7] and is consistent with findings from other researchers [10,12,13].

The SEM analysis provided a more detailed insight into the surface morphology of pulse starch granules, complementing the earlier characterization efforts.

### 3.2. Differential Scanning Calorimetry Properties of Pulse Starches

The temperature parameters To, Tp, and Tc exhibited notable variability among the pulse starches (Table 1 and Table 2), from 62.15 to 74.50, 68.42 to 79.74, and 77.43 to 88.44 °C, respectively. In contrast, the standard starch (corn) displayed 65.17, 69.58, and 77.43 °C values, aligning with established data reported in previous studies [14,15].

The enthalpy (ΔH), serving as a metric for the disruption in the starch double-helical structure, exhibited a range from 6.65 to 12.06 °C for the pulse starches, in contrast to the value of 8.91 °C observed for corn. Black bean showcased the lowest enthalpy (6.65 °C), while Nigerian honey bean (cowpea) showed the highest (12.06 °C). The increased ΔH of Nigerian honey bean (cowpea), 33 °C, suggests a more extensive distribution of double helices and augmented crystallinity, indicative of increased resistance to gelatinization.

Post-gelatinization, the subsequent cooling and storage of the gelatinized pulse starches at 4 °C for 14 days showed the retrogradation of the starch polymers (Table 2), leading to augmented crystallinity and enhanced gel firmness. This degree of retrogradation signifies the resistance of the retrograded pulse starches to digestive enzymes, contributing to a potential reduction in postprandial glucose levels.

The thermal properties of the pulse starches, particularly their higher gelatinization temperatures and enthalpy values, suggest structural robustness contributing to reduced starch digestibility. This resistance to digestion, compounded by retrogradation, could lead to a lower postprandial glucose response, offering potential benefits for managing blood sugar levels.

### 3.3. Molecular Weight Distributions of Amylopectin and Amylose

**Figure 2:**
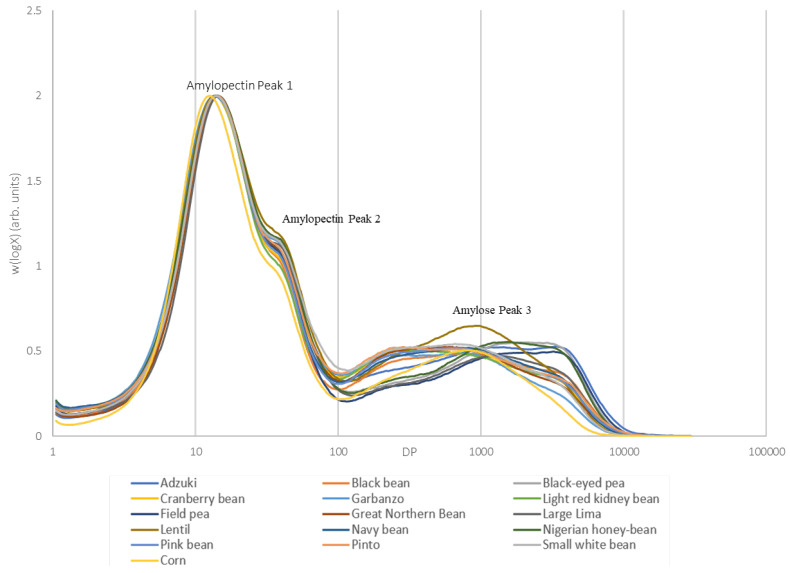
Size exclusion chromatography of linear glucan chains from debranched pulse starches.

The chain length distribution (CLD) of the debranched pulse starch structures, exhibiting amylopectin bimodal peaks followed by amylose, is visually represented in Figure 2. The distribution curves were normalized to the same height as the maximum peak to facilitate comparison, revealing similarities among the CLD curves of the various pulse starch samples. The calculation of the Degree of Polymerization (DP) for amylose and amylopectin, following the methodology outlined by [16], allowed for determining the chain length characteristics.

The DP values were categorized into 1–100 for amylopectin and >100 for amylose [17,18]. The SEC weight CLD showed three peaks, denoting the relative size of the chains within the debranched structure. The first amylopectin peak appeared at DP~13 and the second at DP~37. The first amylopectin peak, representing short A and B1 chains, was indistinguishable across the various pulse starches (Figure 2). The second amylopectin peak, 2, indicating longer internal amylopectin chains, differed somewhat in amounts among the starches. The third peak, 3, representative of amylose, exhibited a broader molecular size range from DP 300 to DP 2300.

Significant differences in amylose branch lengths were observed among the various pulse starches. Light red kidney beans had the shortest amylose branches, with a peak of 369, while field peas had the longest branches, with a peak of 2225. These distinctions in the CLD, particularly concerning the shorter molecules of amylopectin (DP 0–10), longer-chain amylopectin molecules (DP 1–100), and amylose (DP >100), were found to be significantly different among the various pulse starches and corn.

The molecular structure, particularly in terms of the amylose content, has been reported to exhibit a specific relationship with digestibility [19].

### 3.4. In Vitro Digestion of Gelatinized and Retrograded Pulse Starches

#### 3.4.1. Gelatinized and Retrograded Pulse Gels Were Slowly Digestible

In our investigation, starches were derived from adzuki bean, black bean, black-eyed pea, cranberry bean, chickpea, kidney bean, field pea, great northern bean, large lima bean, lentil, navy bean, Nigerian honey bean (cowpea), pink bean, pinto bean, and small white bean. Ref. [7] previously reported on the selection criteria for these pulses, considering starch yield, moisture content, residual protein, and residual lipids.

To determine the digestibility patterns of these pulse starches, we used a methodological approach involving a combination of PAA and rat intestinal acetone powder (RIAP), as reported by [20,21]. At 15 min of incubation with PAA and RIAP, the rate of starch digestion was evaluated. Chickpea starch demonstrated a slower digestion rate (23%), followed by large lima bean (31%), northern bean (32%), and adzuki bean (32%) gels, in contrast to maltose (72%), as illustrated in Table 3.

The digestibility of the retrograded gels after 7 days of cold storage (4 °C) indicates further modulation of the digestion rate. Retrogradation significantly decelerated the digestion rate of the pulse starches at the initial 15 min of PAA and RIAP digestion. This trend was noted in the following order: chickpea (14%), field pea (18%), lentil (18%), navy bean (18%), adzuki bean (19%), large lima bean (26%), and northern bean (27%), compared to maltose (51%) (Table 4). These findings show the distinct impact of retrogradation on the digestibility of the pulse starches, highlighting specific pulses with distinct digestibility profiles. The purified starches varied in their molecular structure, specifically the amylose structure, forming a more compact structure that contributes to less accessibility to digestive enzymes.

#### 3.4.2. Bread Fortification

Compared to traditional white bread, the pulse-fortified bread in our study incorporated 195 g of chickpea flour, representing 13% chickpea starch (Supplementary Table S1). To assess the nutritional impact of these fortifications, we categorized the fortified bread samples based on their percentage digestibility into rapidly digestible starch (RDS), slowly digestible starch (SDS), total digestible starch (TDS), resistant starch (RS), and total starch (TS). Statistical significance (*p* < 0.05) in terms of the RDS content was observed across multiple storage time points (1, 3, 7, and 14 days), with the lowest RDS (37%) on day 1 for the bread fortified with 13% chickpea flour. This significant reduction in RDS content indicates that the fortified bread with chickpea flour exhibited a slower digestibility than the fortified bread with 13% chickpea starch and the traditional white bread (Table 5). Chickpea flour is high in resistant starch and fiber, which can impede the enzymatic breakdown of starch during digestion. Additionally, the protein and fat content in chickpea flour may form complexes with starch, further reducing its availability for rapid digestion. These factors likely contributed to the lower RDS content.

A detailed examination of the SDS content, illustrated in Table 6, revealed an increasing trend as the storage days increased for the bread fortified with 13% chickpea starch. This observed increase in SDS aligns with the rapid retrogradation in chickpea starch, leading to increased crystallinity. This elevated crystallinity subsequently curtailed the activity of pancreatic alpha-amylase and glucosidases, contributing to the increased SDS content over time. Our investigation provides insights into the impact of chickpea flour and chickpea starch fortifications on the digestibility of bread. The observed slower digestibility, notably in the bread fortified with chickpea flour, underscores the potential of these fortifying ingredients to modulate the nutritional profile of baked products. This study offers implications for developing fortified bread with improved digestibility and nutritional characteristics.

## 4. Conclusions

Consuming foods rich in slowly digestible starch (SDS) is essential for enhancing human health due to its association with moderated glucose release and reduced postprandial and insulinemic responses [3]. This study explored pulses as a novel source of SDS, investigating various pulse starches to identify those with the highest content. Building on the work of Sangokunle et al. (2020) [7], we extended our analysis to characterize the surface characteristics of these raw pulse starches.

Purified pulse starches, including chickpea, adzuki bean, great northern bean, large lima bean, black-eyed pea, and Nigeria honey bean (cowpea), exhibit varying digestibility due to distinct amylose retrogradation patterns and elevated amylose contents. Despite criticisms of the Englyst classification of starch into rapidly digestible starch (RDS), SDS, and resistant starch (RS), this framework remains a widely accepted method for analyzing starch digestibility across food matrices. Hence, the Englyst method was used in this study and may be a potential drawback. The use of chickpea starch in our bread study was based on its advantageous slow digestibility profile, which supports the reduction in RDS while increasing SDS and RS, potentially lowering postprandial glycemia. However, we recognize the limitations in our study, including the absence of rheological, textural, sensory, and nutritional analyses, as well as the need for further investigation into the effects of gelation on the textural properties of fortified bread.

This study offers valuable insights for the food industry and lays the groundwork for developing baked products fortified with SDS from pulse starches, specifically chickpea starch, potentially enhancing human health.

## Figures and Tables

**Table 1:** DSC thermal properties of various pulse starches. ^†^ Data with different letters indicate statistical significance.

	Gelatinization			
Type	Onset (°C)	Peak (°C)	Offset (°C)	Enthalpy (J/g)
Adzuki bean	62.15 ± 0.22a ^†^	68.42 ± 0.03a	77.54 ± 0.34a	8.91 ± 1.16ab
Black bean	62.96 ± 1.44ab	71.83 ± 2.64cde	81.04 ± 2.06bc	6.65 ± 3.72a
Black-eyed pea	68.90 ± 0.92e	75.03 ± 1.10f	83.06 ± 0.55c	8.00 ± 0.55ab
Cranberry bean	64.76 ± 0.10bc	71.47 ± 0.51bcde	80.24 ± 1.26abc	9.28 ± 1.78ab
Chickpea	66.48 ± 1.74cd	69.17 ± 0.64ab	77.43 ± 1.47a	11.78 ± 0.96b
Kidney bean	66.19 ± 0.04cd	73.16 ± 0.11def	81.51 ± 1.10bc	8.91 ± 0.98ab
Field pea	68.95 ± 0.03e	73.93 ± 0.17ef	81.83 ± 0.72bc	11.36 ± 0.25ab
Great northern bean	65.90 ± 0.19cd	72.50 ± 0.17def	81.43 ± 1.51bc	8.10 ± 1.44ab
Large lima bean	72.27 ± 0.37f	78.88 ± 1.03g	86.45 ± 1.10d	8.61 ± 2.29ab
Lentil	66.74 ± 0.04d	71.55 ± 0.13bcde	80.14 ± 0.16abc	10.68 ± 1.27ab
Navy bean	65.11 ± 0.13cd	70.96 ± 0.05abcd	79.60 ± 0.33ab	8.33 ± 0.33ab
Nigerian honey bean	74.50 ± 0.11g	79.74 ± 0.15g	88.44 ± 0.43d	12.06 ± 0.61b
Pink bean	66.18 ± 0.06cd	72.35 ± 0.32de	81.01 ± 0.35bc	7.69 ± 0.38ab
Pinto bean	65.84 ± 0.45cd	71.29 ± 0.84bcd	79.96 ± 1.66abc	8.50 ± 2.26ab
Small white bean	65.88 ± 0.39cd	72.29 ± 0.33de	82.05 ± 0.72bc	8.75 ± 1.06ab
Corn	65.17 ± 0.39cd	69.58 ± 0.72abc	77.43 ± 0.74a	8.91 ± 2.01ab

**Table 2:** DSC thermal properties of retrograded pulse starches after 7 days of storage at 4 °C. ^†^ Data with different letters indicate statistical significance.

	Retrogradation (7 days, 4 °C)			
Type	Onset (°C)	Peak (°C)	Offset (°C)	Enthalpy (J/g)
Adzuki bean	48.12 ± 0.23	68.42 ± 0.03a ^†^	77.54 ± 0.34a	3.04 ± 0.59
Black bean	47.69 ± 2.44	71.83 ± 2.64cde	81.04 ± 2.06bc	2.76 ± 1.19
Black-eyed pea	46.44 ± 1.44	75.03 ± 1.10f	83.06 ± 0.55c	5.52 ± 0.42
Cranberry bean	47.07 ± 0.24	71.47 ± 0.51bcde	80.24 ± 1.26abc	5.24 ± 0.65
Chickpea	46.62 ± 0.66	69.17 ± 0.64ab	77.43 ± 1.47a	5.81 ± 0.54
Kidney bean	46.36 ± 0.26	73.16 ± 0.11def	81.51 ± 1.10bc	8.01 ± 0.65
Field pea	46.61 ± 0.07	73.93 ± 0.17ef	81.83 ± 0.72bc	8.46 ± 0.44
Great northern bean	46.89 ± 0.35	72.50 ± 0.17def	81.43 ± 1.51bc	3.04 ± 0.59
Large lima bean	48.87 ± 0.47	78.88 ± 1.03g	86.45 ± 1.10d	6.92 ± 1.21
Lentil	49.53 ± 0.90	71.55 ± 0.13bcde	80.14 ± 0.16abc	6.04 ± 1.47
Navy bean	48.61 ± 0.45	70.96 ± 0.05abcd	79.60 ± 0.33ab	7.31 ± 1.30
Nigerian honey bean	50.15 ± 0.37	79.74 ± 0.15g	88.44 ± 0.43d	6.10 ± 0.52
Pink bean	50.49 ± 1.91	72.35 ± 0.32de	81.01 ± 0.35bc	6.72 ± 0.74
Pinto bean	49.18 ± 1.33	71.29 ± 0.84bcd	79.96 ± 1.66abc	8.30 ± 0.31
Small white bean	49.44 ± 0.76	72.29 ± 0.33de	82.05 ± 0.72bc	6.79 ± 1.74
Corn	47.38 ± 0.82	69.58 ± 0.72abc	77.43 ± 0.74a	5.49 ± 0.49

**Table 3:** Percentage digestibility of gelatinized pulse starches compared to maltose using RIAP and alpha-amylase.

	Time (min)					
Starches	0	15	30	45	60	120
Adzuki bean	1.0 ± 1.2	33.2 ± 8.4	60.4 ± 8.1	79.4 ± 6.9	92.2 ± 4.9	109.4 ± 5.3
Black bean	0.9 ± 1.3	36.4 ± 10.8	65.0 ± 7.6	76.7 ± 4.0	86.1 ± 6.0	105.9 ± 1.5
Black-eyed pea	1.0 ± 1.4	48.1 ± 8.6	79.4 ± 2.9	90.1 ± 4.4	104.4 ± 8.2	119.8 ± 7.4
Cranberry bean	1.2 ± 1.2	35.8 ± 6.5	61.9 ± 3.2	78.5 ± 6.9	86.9 ± 6.8	102.0 ± 11.3
Chickpea	1.0 ± 1.2	24.6 ± 0.2	55.7 ± 5.3	75.0 ± 7.0	86.5 ± 8.6	103.8 ± 6.6
Kidney bean	0.8 ± 1.3	41.5 ± 9.5	67.8 ± 2.2	81.0 ± 5.4	92.9 ± 5.4	109.8 ± 9.9
Field pea	0.9 ± 1.4	44.2 ± 8.5	68.0 ± 2.1	82.6 ± 8.1	95.4 ± 8.4	109.3 ± 8.7
Great northern bean	−0.2 ± 1.4	71.1 ± 17.0	74.9 ± 4.7	81.3 ± 10.2	90.1 ± 8.5	105.0 ± 7.2
Large lima bean	0.7 ± 1.3	32.7 ± 7.3	55.6 ± 11.3	63.5 ± 10.4	70.5 ± 14.2	91.5 ± 14.6
Lentil	0.9 ± 1.3	42.0 ± 0.7	65.3 ± 5.0	79.4 ± 5.5	89.1 ± 7.8	107.9 ± 13.0
Navy bean	0.8 ± 1.3	43.7 ± 4.0	68.0 ± 8.2	85.9 ± 5.1	93.1 ± 9.6	109.5 ± 9.1
Nigerian honey bean	0.9 ± 1.4	53.3 ± 10.5	71.8 ± 9.1	86.0 ± 8.5	94.5 ± 6.3	114.4 ± 9.4
Pink bean	0.9 ± 1.3	54.2 ± 5.9	77.0 ± 6.3	87.8 ± 9.3	94.7 ± 11.8	108.7 ± 9.7
Pinto bean	0.8 ± 1.3	51.7 ± 1.6	74.7 ± 5.7	85.9 ± 7.6	92.1 ± 9.4	109.1 ± 8.7
Small white bean	0.8 ± 1.3	50.0 ± 3.4	73.6 ± 10.0	80.8 ± 7.2	95.7 ± 10.8	113.0 ± 9.9
Maltose	−1.1 ± 1.6	70.4 ± 7.7	94.9 ± 11.8	101.9 ± 13.0	107.5 ± 11.3	116.4 ± 18.7

**Table 4:** Percentage digestibility of retrograded pulse starches compared to maltose using RIAP and alpha-amylase.

	Time (min)					
Starches	0	15	30	45	60	120
Adzuki bean	0.0 ± 0.1	25.5 ± 2.7	53.1 ± 1.1	66.1 ± 4.1	85.4 ± 9.0	107.0 ± 16.4
Black bean	0.0 ± 0.0	41.3 ± 1.1	57.4 ± 4.9	74.2 ± 2.9	85.2 ± 3.5	93.9 ± 7.4
Black-eyed pea	0.0 ± 0.0	45.3 ± 5.0	74.3 ± 3.2	89.1 ± 3.0	95.3 ± 2.9	116.6 ± 3.5
Cranberry bean	0.0 ± 0.0	46.5 ± 3.5	63.0 ± 0.5	83.8 ± 4.8	90.7 ± 6.2	101.8 ± 11.5
Chickpea	0.0 ± 0.1	19.3 ± 1.0	50.6 ± 3.5	66.4 ± 1.2	78.4 ± 5.5	95.9 ± 9.1
Kidney bean	0.0 ± 0.0	40.2 ± 6.6	56.8 ± 12.4	77.1 ± 7.9	87.1 ± 5.6	102.5 ± 10.8
Field pea	0.0 ± 0.2	24.7 ± 2.5	50.2 ± 2.5	70.0 ± 1.9	85.2 ± 0.3	112.2 ± 7.9
Great northern bean	0.0 ± 0.6	41.1 ± 4.5	59.3 ± 5.2	81.5 ± 12.2	87.6 ± 13.2	106.4 ± 18.3
Large lima bean	0.0 ± 0.0	35.5 ± 1.8	60.5 ± 1.8	75.2 ± 0.6	83.9 ± 1.9	108.8 ± 2.3
Lentil	0.0 ± 0.1	25.4 ± 2.0	45.0 ± 3.2	58.4 ± 7.1	73.9 ± 4.0	93.8 ± 19.4
Navy bean	0.0 ± 0.0	24.6 ± 3.9	45.6 ± 3.5	63.2 ± 1.9	73.5 ± 2.6	91.4 ± 11.2
Nigerian honey bean	0.0 ± 0.0	51.1 ± 3.7	71.4 ± 2.6	82.5 ± 5.1	86.5 ± 4.6	103.9 ± 1.8
Pink bean	0.0 ± 0.5	44.7 ± 0.7	65.4 ± 4.8	76.3 ± 6.1	83.2 ± 8.4	104.4 ± 10.7
Pinto bean	0.0 ± 0.0	39.6 ± 2.5	60.1 ± 1.4	72.0 ± 4.4	81.2 ± 4.2	94.7 ± 7.3
Small white bean	0.0 ± 0.1	46.9 ± 1.0	67.4 ± 3.6	77.5 ± 5.2	84.1 ± 6.1	103.9 ± 11.0
Maltose	0.0 ± 0.3	69.1 ± 2.4	94.0 ± 6.8	106.8 ± 8.9	110.4 ± 12.0	122.7 ± 12.4

**Table 5:** SDS and RS content of bread fortified with chickpea starch during storage (days 1 and 3). Data are reported as average and the standard deviation of the mean (triplicate). WB, white bread; GS, chickpea starch; GF, chickpea flour. ^†^ Data with different letters indicate statistical significance.

	Day 1			Day 3		
Starches (g/100 g)	WB	GS	GF	WB	GS	GF
RDS	43.5 ± 1.3a ^†^	42.5 ± 0.7a	36.6 ± 1.4b	48.7 ± 0.8a	44.0 ± 1.0b	40.0 ± 1.2b
SDS	2.0 ± 0.2a	3.2 ± 0.1ab	4.7 ± 0.9b	1.8 ± 0.9	7.5 ± 0.8b	1.8 ± 1.1a
TDS	45.1 ± 0.8	48.8 ± 1.1	44.8 ± 1.2	52.0 ± 0.1b	52.5 ± 0.0b	46.5 ± 0.6a
RS	1.3 ± 0.0a	1.85 ± 0.0b	1.5 ± 0.1c	2.0 ± 0.1a	2.4 ± 0.0b	1.9 ± 0.0a
TS	48.0 ± 1.3	50.6 ± 1.1	46.3 ± 1.3	54.0 ± 0.2a	55.0 ± 0.0a	48.5 ± 0.6b

**Table 6:** SDS and RS content of bread fortified with chickpea starch during storage (days 7 and 14). Data are reported as average and the standard deviation of the mean (triplicate). WB, white bread; GS, chickpea starch; GF, chickpea flour. ^†^ Data with different letters indicate statistical significance.

	Day 7			Day 14		
Starches (g/100 g)	WB	GS	GF	WB	GS	GF
RDS	50.6 ± 1.7b ^†^	46.8 ± 1.0b	41.0 ± 1.2a	49.0 ± 0.9a	43.8 ± 1.0b	38.8 ± 0.3b
SDS	1.0 ± 1.6a	9.3 ± 0.8b	6.5 ± 0.5b	4.3 ± 0.3a	9.1 ± 0.8b	9.5 ± 0.2c
TDS	51.4 ± 0.0a	55.3 ± 0.1b	46.2 ± 1.1c	53.2 ± 0.8	51.3 ± 1.5	45.3 ± 3.6
RS	2.0 ± 0.0a	2.6 ± 0.0b	2.0 ± 0.0c	2.2 ± 0.1b	2.7 ± 0.1a	2.1 ± 0.0b
TS	53.4 ± 0.0a	57.9 ± 0.1b	48.1 ± 1.1c	55.4 ± 0.9	54.0 ± 1.6	103.8 ± 6.6

## Data Availability

The original contributions presented in this study are included in the article/Supplementary Materials. Further inquiries can be directed to the corresponding authors.

## Supplementary Materials

The following supporting information can be downloaded at: https://www.mdpi.com/article/10.3390/foods14020328/s1, Figure S1: Gelatinization and retrogradation characteristics of purified pulse starches compared to corn starch; Table S1: Recipe for American sandwich bread fortified with 13% purified chickpea starch.
