# Supplementary material for: Characterization and In Vitro Digestion Kinetics of Purified Pulse Starches: Implications on Bread Formulation
Source: Foods. 2025 Jan 20;14(2):328. doi: 10.3390/foods14020328 (PMC11765446; doi:10.3390/foods14020328)
Supplement: Supplementary file 1 [file foods-14-00328-s001.zip › foods-3363660-supplementary.pdf]

Optional supplementary information

Table S1: Recipe for American sandwich bread fortified with 13% purified chickpea starch.

**Bread Recipe**		
**Ingredients**	**Recipe 001**	**Recipe 002**
Whole wheat bread flour	195 g	195 g
Chickpea flour	195g	NA
Chickpea starch	NA	13 % of the total weight of the recipe
Yeast	7 g	7 g
Salt	8.54 g	8.5 g
Whole milk	6 oz	6 oz
Water	2.7 oz	2.7 oz
Unsalted butter	28.4 g	28.4 g
Honey	2 tbsp	2 tbsp
Makes: 1 loaf		
Rising time: 3 h		
Baking time: 40 min		
Cooling time: 45 min – 1 h at room temperature		

Recipes were developed from Bread Illustrated, a step-by-step guide to achieving bakery-quality results at home—America's Test Kitchen.

Figure S1: Gelatinization and retrogradation characteristics of purified pulse starches compared to corn starch

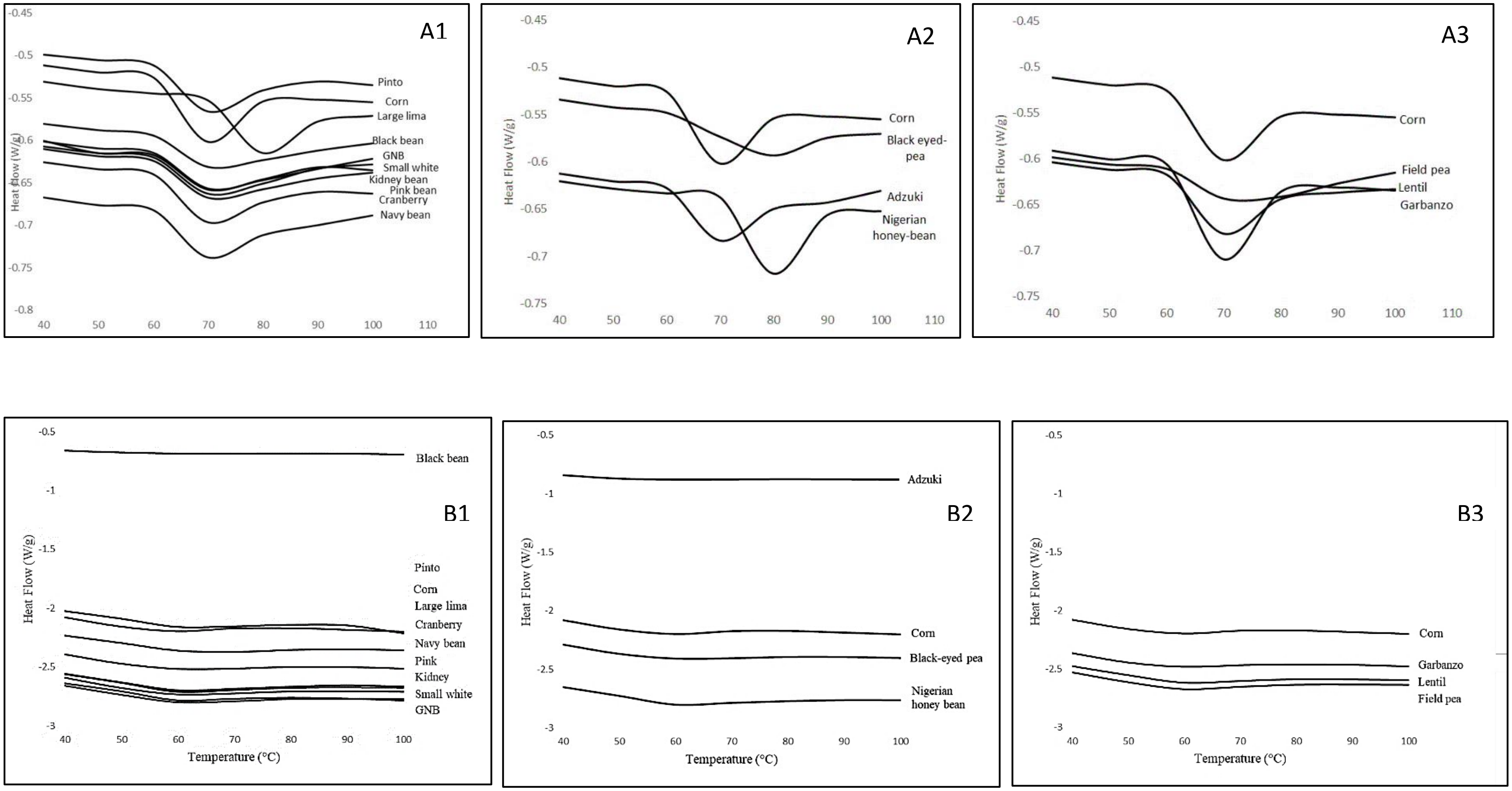

Gelatinization (A1-A3) and retrogradation (B1-B2) thermographs of purified pulse starches.
